# Down-Regulation of mir-424 Contributes to the Abnormal Angiogenesis via MEK1 and Cyclin E1 in Senile Hemangioma: Its Implications to Therapy

**DOI:** 10.1371/journal.pone.0014334

**Published:** 2010-12-14

**Authors:** Taiji Nakashima, Masatoshi Jinnin, Tomomi Etoh, Satoshi Fukushima, Shinichi Masuguchi, Keishi Maruo, Yuji Inoue, Tsuyoshi Ishihara, Hironobu Ihn

**Affiliations:** Department of Dermatology and Plastic Surgery, Faculty of Life Sciences, Kumamoto University, Kumamoto, Japan; Tufts University, United States of America

## Abstract

**Background:**

Senile hemangioma, so-called cherry angioma, is known as the most common vascular anomalies specifically seen in the aged skin. The pathogenesis of its abnormal angiogenesis is still unclear.

**Methodology/Principal Findings:**

In this study, we found that senile hemangioma consisted of clusters of proliferated small vascular channels in upper dermis, indicating that this tumor is categorized as a vascular tumor. We then investigated the mechanism of endothelial proliferation in senile hemangioma, focusing on microRNA (miRNA). miRNA PCR array analysis revealed the mir-424 level in senile hemangioma was lower than in other vascular anomalies. Protein expression of MEK1 and cyclin E1, the predicted target genes of mir-424, was increased in senile hemangioma compared to normal skin or other anomalies, but their mRNA levels were not. The inhibition of mir-424 in normal human dermal microvascular ECs (HDMECs) using specific inhibitor *in vitro* resulted in the increase of protein expression of MEK1 or cyclin E1, while mRNA levels were not affected by the inhibitor. Specific inhibitor of mir-424 also induced the cell proliferation of HDMECs significantly, while the cell number was decreased by the transfection of siRNA for MEK1 or cyclin E1.

**Conclusions/Significance:**

Taken together, decreased mir-424 expression and increased levels of MEK1 or cyclin E1 in senile hemangioma may cause abnormal cell proliferation in the tumor. Senile hemangioma may be the good model for cutaneous angiogenesis. Investigation of senile hemangioma and the regulatory mechanisms of angiogenesis by miRNA in the aged skin may lead to new treatments using miRNA by the transfection into senile hemangioma.

## Introduction

Mature blood vessels are composed of two distinct cell types: a continuous monolayer of endothelial cells (ECs) forming the inner surface of the vessel wall and an outer layer of perivascular supporting cells including pericytes and smooth muscle cells [1]. On the other hand, the term ‘vascular anomalies’ generically indicates various conditions including developmental error or dysregulated developmental processes of vascular morphogenesis. According to a classification proposed by Mulliken and Glowacki in 1982 and 1996, cutaneous vascular anomalies can be divided into vascular tumor characterized by cellular hyperplasia (too many normal cells), and vascular malformations characterized by enlargement of dysplastic vessels [2]. Vascular tumors include infantile hemangioma, kaposiform hemangioendothelioma, and tufted angioma. Vascular malformations are further classified into capillary, venous, lymphatic, and arteriovenous malformations. Malignant vascular tumors such as angiosarcoma or Kaposi's sarcoma were not included in this classification.

Senile hemangioma, so-called cherry angioma, is a smooth reddish dome-shaped tumor, mainly found on the trunk of the elderly person [3]. A venous lake is also smooth dark bluish dome-shaped papule/nodule that appears on the lower lip, face and ears [4]. They are known as the most common vascular anomalies specifically seen in the aged skin. These tumors are usually asymptomatic, but sometimes become problematic because of bleeding and disfigurement. However, there have been few therapeutic options, such as surgical resection or laser treatments, in spite of recent advances in the development of anti-angiogenic therapies against various vascular anomalies [5]–[7].

These tumors are not described in the above classification system, and the pathogenesis of these tumors has been poorly investigated. Venous lake is frequent in lower lip, indicating the correlation with sunlight [8], [9]. On the other hand, senile hemangioma is not likely to be associated with UV exposure because of their distribution on the trunk. Tuder et al. reported that senile hemangiomas are overgrowths made up of ECs with terminal differentiation, based on the low immunoreactivity of tumor ECs with Ki-67 and activation-related antibody in vivo and in vitro [10]. Thus, the tumor is thought to have different etiology from abnormal angiogenesis seen in intrinsic aged skin or photoaged skin, which is characterized by an age-dependent reduction of cutaneous microvasculature [11], [12].

In this study, we aimed to clarify the pathogenesis of these tumors. First, we tried to characterize these tumors based on the above classification system, and presented that senile hemangioma is vascular tumor and venous lake is vascular malformation. We then investigated the mechanism(s) underlying the abnormally increased endothelial proliferation in senile hemangioma, focusing on microRNA (miRNA). miRNAs, short ribonucleic acid molecules on average only 22 nucleotides long, are post-transcriptional regulators that bind to complementary sequences in the three prime untranslated regions (3′ UTRs) of mRNAs, leading to gene silencing. There are thought to be more than 1000 miRNAs in the human genome, which may target about 60% of mammalian genes [13]. Recent vigorous efforts of research in this field indicated that miRNAs play a role in angiogenesis as well as immune response or carcinogenesis in vivo [14]–[18]. Our study demonstrated a regulatory mechanism of angiogenesis in the aged skin by miRNAs.

## Materials and Methods

### Patient material and Ethics Statement

This research was approved by the Ethics Review Committee in Kumamoto University (No. 177). Skin specimens were obtained from 7 senile hemangioma, 3 venous malformation, 4 angiosarcoma, 4 venous lake, and 3 infantile hemangioma (Table 1). Seven control skin samples are obtained from routinely discarded skin of healthy human subjects undergoing skin graft. Control and patient samples were collected and processed immediately after resection in parallel. Written informed consent was obtained according to the Declaration of Helsinki.

**Table 1:** Clinical data: ages of patients at the time of resection and location of the lesions.

		sex	age	location
senile hemangioma	1	M	79	trunk
	2	M	60	neck
	3	M	45	upper extremity
	4	M	75	trunk
	5	M	57	upper extremity
	6			trunk
	7	F	69	face
venous malformaiton	1	F	66	upper extremity
	2	F	78	face
	3	M	50	upper extremity
angiosarcoma	1	F	77	head
	2	M	80	head
	3	M	86	head
	4	M	74	head
venous lake	1	M	55	lip
	2	F	63	lip
	3	M	54	cheek
	4	F	44	lip
infantile hemangioma	1	F	0	lower extremity
	2	M	2	upper extremity
	3	F	3	face

Sample No.5 and No.6 of senile hemangioma were obtained from same patient.

### RNA isolation and quantitative real-time polymerase chain reaction (PCR)

Skin specimens were obtained from 7 senile hemangioma, 3 venous malformation, 4 angiosarcoma, 4 venous lake, 3 infantile hemangioma and 7 healthy controls. They were fixed in 10% neutral-buffered formalin, embedded in paraffin, and sliced. Total RNA isolation from paraffin-embedded section of normal skin and tumor tissues were performed with RNeasy FFPE kit (Qiagen, Valencia, CA) following the manufacturer's instructions. cDNA was synthesized from total RNA with PrimeScript RT rea(Takara Bio Inc, Shiga, Japan). Quantitative real-time PCR with a Takara Thermal Cycler Dice (TP800)® used primers and templates mixed with the SYBR Premix Ex gent Kit TaqII (Takara Bio Inc). Primer sets for vascular endothelial growth factor receptor (VEGFR) 1, VEGFR2 and GAPDH were purchased from SA Biosciences (Frederick, MD). Primers for HIF-1α, MEK1 and cyclin E1 were from Takara. These primer sets were prevalidated to generate single amplicons. MEK1 was amplified for 50 cycles of denaturation for 5s at 95°C, annealing for 30s at 70°C, whereas annealing temperature was set at 60°C for the other primers. Data generated from each PCR reaction were analyzed using Thermal Cycler Dice Real Time System ver2.10B (Takara Bio Inc). Specificity of reactions was determined by melting curve analysis. Transcript levels were normalized to GAPDH.

### miRNA extraction and PCR array analysis of miRNA expression

Small RNAs were extracted using a miRNeasy FFPE kit (Qiagen). Then, RNAs were reverse-transcribed into first strand cDNA using an RT^2^ miRNA First Strand Kit (SABiosciences, Frederick, MD). For RT^2^ Profiler PCR Array (SABioscience), the cDNA was mixed with RT^2^ SYBR Green/ROX qPCR Master Mix and the mixture was added into a 96-well RT^2^ miRNA PCR Array (SABiosciences) that included primer pairs for 88 human miRNAs. PCR was performed on a Takara Thermal Cycler Dice (TP800®) following the manufacture's protocol. Threshold cycle (Ct) for each miRNA was extracted using Thermal Cycler Dice Real Time System ver2.10B. The raw C_t_ was normalized using the values of small RNA housekeeping genes.

For quantitative real-time PCR, primers for mir-424, mir-1 or U6 (SABioscience) and templates were mixed with the SYBR Premix Ex TaqII (Takara Bio Inc). DNA was amplified for 50 cycles of denaturation for 5s at 95°C, annealing for 30s at 60°C. Data generated from each PCR reaction were analyzed using Thermal Cycler Dice Real Time System ver2.10B (Takara Bio Inc). Transcript levels were normalized to U6.

### Immunohistochemical staining

Paraffin sections were deparaffinized in xylen and rehydrated in a graded ethanol series. Antigens were retrieved by incubation with citrate buffer pH 6 for 5 min with microwave oven. Endogenous peroxidase activity was inhibited, after which sections were incubated with 5% milk for 30min and then reacted with the antibodies for α-smooth muscle actin or type IV collagen (Abcam, Cambridge, MA, 1∶300) overnight at 4°C. After excess antibody was washed out with PBS, samples were incubated with HRP-labeled goat anti-mouse antibody (Nichirei, Tokyo, Japan) for 60min. The reaction was visualized by the diaminobenzidine substrate system (Dojin, Kumamoto, Japan). Slides were counterstained with Mayer's haematoxylin, and examined under a light microscope (OLYMPUS BX50, Tokyo, Japan).

### *In situ* hybridization

In situ hybridization was performed with 5′-locked digoxigenin-labeled nucleic acid (LNA) probes complementary to human mature mir-424 and scrambled negative control (Exiqon, Vedbaek, Denmark) [19], [20]. Briefly, human tissues were deparaffinized and deproteinized with protease K for 5 min. Slides were then washed in 0.2% Glycine in PBS and fixed with 4% paraformaldehyde. Hybridization was performed at 48°C overnight followed by blocking with 2% fetal bovine serum and 2% bovine serum albumin in PBS and 0.1% Tween 20 (PBST) for 1 hour. The probe-target complex was detected immunologically by a digoxigenin antibody conjugated to alkaline phosphatase acting on the chromogen nitro blue tetrazolium/5-bromo-4-chloro-3-indolyl phosphate (Roche Applied Science, Mannheim, Germany). Slides were counterstained with nuclear fast red, and examined under a light microscope (OLYMPUS BX50; Tokyo, Japan).

### Immunofluorescence

Paraffin sections were deparaffinized in xylen and rehydrated in a graded ethanol series. Antigens were retrieved by incubation with 0.1% trypsin at 37°C for 5 min. The slides were permeabilized in 0.5% Triton-PBS for 5min and blocked in 5% nonfat dry milk for 30min at room temperature. As the primary antibodies, rabbit anti-MEK1 (1∶50, Santa Cruz Biotechnology) or rabbit anti-cyclin E1 (1∶50, Santa Cruz Biotechnology) with mouse anti-CD34 (1∶25, DakoCytomation, Carpinteria, CA) diluted in 5% milk in PBS, were applied to the sections. The sections were incubated overnight at 4°C, followed by PBS-0.05% Triton X-100 washes. Matching isotype IgG was used as a negative control. Then, Alexa Fluor 488 anti-rabbit (1∶200, Invitrogen, Carlsbad, CA) and Rhodamine-conjugated anti-mouse secondary antibodies (1∶100, Jackson ImmunoResearch, Suffolk, UK) were applied to the sections. After 1 hour at room temperature, sections were washed and mounted with VECTASHIELD mounting medium with DAPI (Vector, Burlingame, CA). Zeiss Axioskop 2 microscope (Carl Zeiss, Oberkochen, Germany) was used for fluorescence microscopy.

### Cell cultures

Human dermal microvascular ECs (HDMECs, CC-2543) were obtained from Lonza (Walkersville, MD). HDMECs were grown on 0.2% gelatin-coated dishes in EGM-2 (Clonetics, San Diego, CA) with 20% fetal bovine serum (Hyclone, Logan, UT) and Antibiotic-Antimycotic (Invitrogen, Carlsbad, CA) in 5% CO_2_ at 37°C [21], [22]. Cells were passaged 1∶3 every 4 to 6 days and used between passages 3 to 9.

### The transient transfection

siRNAs against MEK1 or cyclin E1 was purchased from Santa Cruz Biotechnology. miRNA inhibitors were from Qiagen. Lipofectamine RNAiMAX (Invitrogen, Carlsbad, CA) was used as transfection reagent. For reverse transfection, siRNA (6pmol) or miRNA inhibitor (60pmol) mixed with transfection reagent were added when cells were plated, followed by incubation for 48–72 hours at 37°C in 5% CO_2_. Control experiments showed transcript levels for target genes or miRNAs to be reduced by >80% (data not shown).

### Cell lysis and immunoblotting

HDMEC were washed with cold PBS twice and lysed in lysis buffer (Denaturin Cell Extraction Buffer, BIOSOURCE, Camarillo, CA). Aliquots of cell lysates (normalized for protein concentrations as measured by the Bio-Rad reagent) were separated on sodium dodecyl sulfate-polyacrylamide gels and transferred to PVDF membranes. The membranes were blocked for 1 hour and incubated overnight at 4°C with antibody for MEK1 (Santa Cruz Biotechnology, Santa Cruz, CA) or cyclin E1 (Santa Cruz Biotechnology). The membranes were washed in Tris-buffered saline (TBS) and 0.1% Tween 20, incubated with secondary antibody, and washed again. The detection was performed using the ECL system (Amersham, Arlington Heights, IL) according to the manufacturer's recommendations. As a loading control, immunoblotting was also performed using antibodies against β-actin.

### Cell count

HDMECs were detached from the wells by trypsin treatment and counted using a Coulter® Particle Counter (Beckman Coulter, Fullerton, CA) [22], [23].

### Statistical analysis

Statistical analysis was carried out with the Mann-Whitney test for comparison of means. P values less than 0.05 were considered significant.

## Results

### Pathologic features of senile hemangioma and venous lake

**Figure 1:**
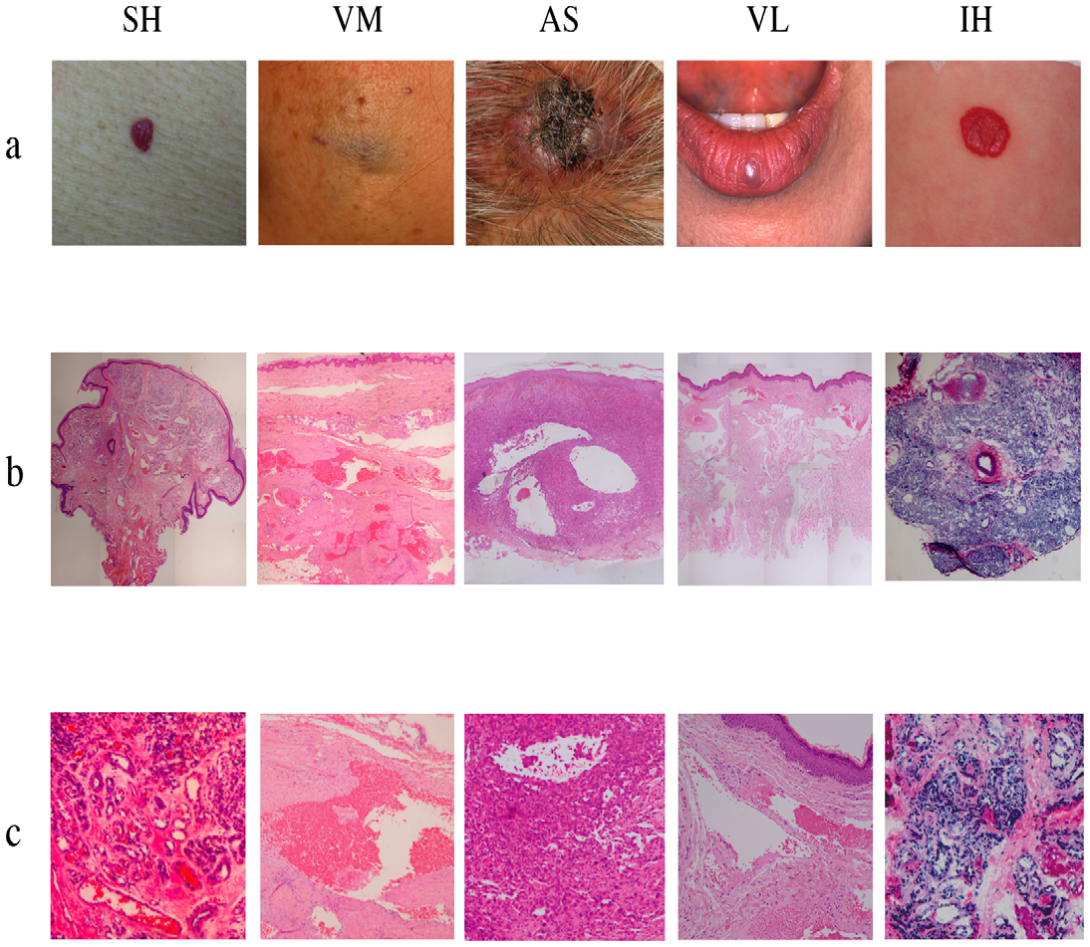
Clinical presentation and histological findings of vascular anomalies. Panel (a) shows clinical pictures of senile hemangioma (SH), vascular malformation (VM), angiosarcoma (AS), venous lake (VL) and infantile hemangioma (IH). Panel (b) (magnification, ×10) and (c) (magnification, ×100) show hematoxylin-eosin staining of each anomalies. SH showed clusters of proliferated small vascular channels in upper dermis and dilated vessels in the deep dermis. AS consisted of diffuse proliferation of atypical ECs accompanied with irregular vessel-like spaces. Diffuse proliferation of tumor cells and dilated vessels were seen in IH. VM and VL were characterized by thin-walled, dilated vessels accompanied with thrombosis throughout the dermis.

As shown in Fig. 1, histologically, senile hemangioma showed clusters of proliferated small vascular channels in upper dermis. Some of the vessels were dilated, especially in the deep dermis. The overall appearance with proliferative nature of ECs was also seen in infantile hemangioma and angiosarcoma but not in venous malformation. On the other hand, histopathologic examination of venous lake revealed thin-walled, dilated vessels accompanied with thrombosis throughout the dermis, which is similar to venous malformation. Thus, senile hemangioma is likely to be categorized as vascular tumor, whereas venous lake seems to be one of the vascular malformations.

### The expression of angiogenic factors or the structure of vessels are maintained in senile hemangioma and venous lake

**Figure 2:**
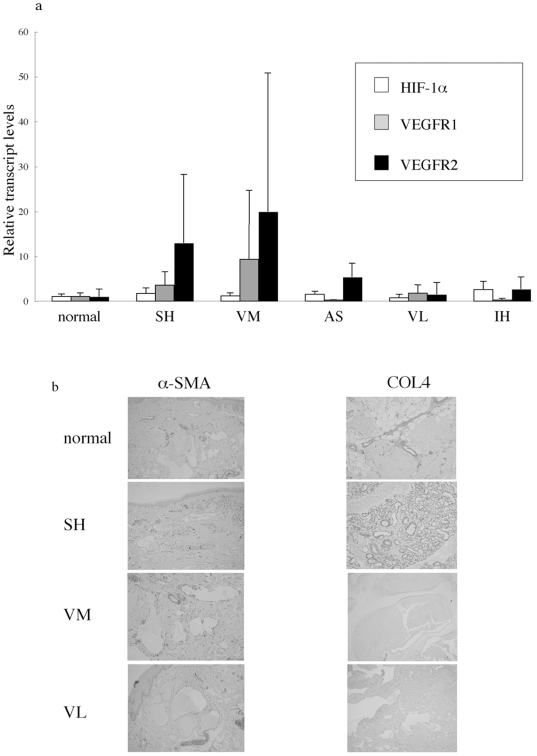
The expression of angiogenic factors or the structure of vessels. (a) Mean relative transcript levels of HIF-1α, VEGFR1 or VEGFR2 (normalized to GAPDH) in tissues from 7 normal skin (normal) or vascular anomalies such as 7 senile hemangioma (SH), 3 vascular malformation (VM), 4 angiosarcoma (AS), 4 venous lake (VL) and 3 infantile hemangioma (IH) by real-time quantitative PCR. The transcript levels in samples of normal skin were set at 1. Error bars represent SD of +1. (b) The expression of α-smooth muscle actin (SMA) or type IV collagen (COL4) in the affected vessels of senile hemangioma (SH), vascular malformation (VM) and venous lake (VL). Paraffin sections were subjected to immunohistochemical analysis with antibodies against α-SMA or COL4 as described in ‘Materials and Methods’. Results are representative of several cases.

Next, we tried to characterize these vascular anomalies further. Up-regulation of HIF-1α and down-regulation of VEGFR1 is reported to be characteristic to infantile hemangioma while down-regulation of VEGFR1 is also seen in angiosarcoma, and such abnormal expression of angiogenic factors are thought to play some role in their pathogenesis [21], [24], [25]. Based on the common feature of senile hemangioma with infantile hemangioma or angiosarcoma as described above, we compared the expression levels of these factors by real-time PCR using total RNA derived from sections of normal skin and various vascular anomalies (Fig. 2a). Although the mRNA levels of HIF-1α, VEGFR1 and VEGFR2 were increased in SH tissue and were not increased in VL tissue compared with those in normal skin, the values were quite variable and there was no significant difference between senile hemangiomas or venous lake and normal skin or other vascular anomalies. Thus, these angiogenic factors are not likely to correlate with the formation of senile hemangioma and venous lake.

Also, we determined whether the structure of vessels including smooth muscle cells and the basement membranes is normally maintained in senile hemangioma or venous lake. Immunohistochemical staining showed no abnormality in the expression and distribution of α-smooth muscle actin (SMA) and type IV collagen on the proliferated small capillaries of senile hemangioma, while α-SMA expression in venous malformation partly disappeared and became irregular, causing dilated vessels (Fig. 2b), as described previously [26]. To note, most of the affected vessels of venous lake, which has similar dilated vessels to venous malformation, showed intact α-SMA expression. These results indicate that angiogenic factors or vessel structures are not likely to be associated with the formation of senile hemangioma and venous lake. Although venous lake is similar to venous malformation histologically, the etiology seems to be different.

### miRNA expression profile in senile hemangioma

**Figure 3:**
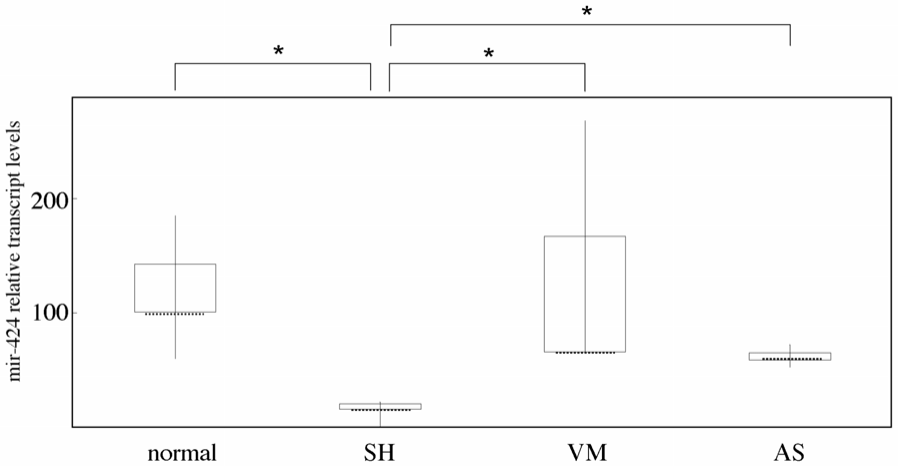
The transcript mir-424 level in senile hemangioma. Box-and-whisker plots of mir-424 levels in normal skin (normal), senile hemangioma (SH), vascular malformation (VM) and angiosarcoma (AS) by real-time PCR. Boxes represent the interquartile ranges and the lines emanating from each box (the whiskers) extend to the smallest and largest observations in a group. Dotted bars show medians. The median transcript level in samples of normal skin was set at 100. * P<0.05 as compared with the values in normal skin, VM or AS.

**Figure 4:**
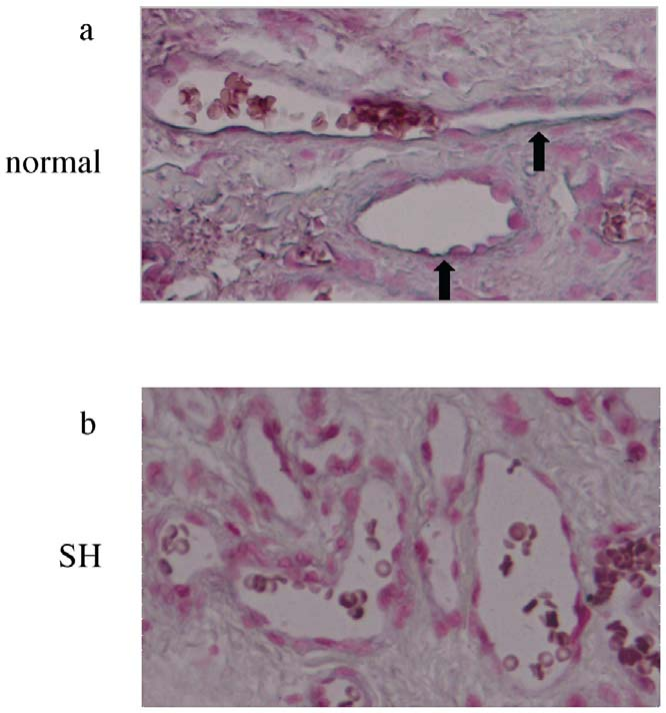
The immunoreactivity for mir-424 in senile hemangioma. In situ detection of mir-424 in paraffin-embedded, formalin-fixed tissues of normal skin (a) and senile hemangioma (SH, b). Nucleus was counterstained with nuclear fast red (magnification, ×200). The immunoreactivity for mir-424 (blue) is indicated by arrows. Results are representative of 3 normal skins and 3 SH.

Then, we examined why the ECs of senile hemangioma are proliferated. We expected miRNA as the angiogenic factor which induce the proliferation of ECs in senile hemangioma. A mixture of equal amounts of miRNAs from 3 senile hemangioma, 3 venous malformation or 3 angiosarcoma were prepared, and miRNA expression profile in each tumor in vivo was evaluated using miRNA PCR array, consisting of 88 miRNAs involved in human cell differentiation and development (Table 2). There were several overexpressed or suppressed miRNAs specifically in senile hemangioma. Among them, the expression of mir-424 in senile hemangioma was decreased compared to that in venous malformation (2-cycle difference in ΔΔCT method) or angiosarcoma (1-cycle). Real-time PCR using specific primer for mir-424 with increased number of samples (7 senile hemangioma, 3 venous malformation and 4 angiosarcoma) revealed that the mir-424 level in senile hemangioma was further lower than normal skin or other vascular anomalies (Fig. 3). In addition, in situ hybridization showed that signal for mir-424 was evident in normal vessels, but not in the ECs of senile hemangioma (Fig. 4). To note, we determined mir-424 expression in skin samples from young and old healthy controls, but there was no significant difference between young and old skin (data not shown). These results suggest that decreased mir-424 level in vivo is specific to senile hemangioma.

**Table 2:** The expression profiles of miRNAs as measured with the PCR array.

ΔΔCt	Senile hemangioma	Venous Malformation	Angiosarcoma
let-7a	−1.62	1.16	−0.58
let-7b	−0.66	1.12	0.66
let-7c	2.95	4.34	2.93
let-7d	2.59	1.73	6.79
let-7e	1.26	3.34	2.35
let-7f	6.92	5.77	10.52
let-7g	2.40	3.17	2.29
let-7i	2.56	2.17	2.88
miR-1	7.20	2.67	5.67
miR-7	6.87	5.89	5.29
miR-9	5.76	5.01	6.66
miR-10a	−0.38	2.76	2.12
miR-10b	−1.11	−0.16	0.62
miR-15a	5.35	4.27	5.91
miR-15b	1.31	3.57	1.18
miR-16	−0.44	−1.58	0.00
miR-17	3.40	2.34	4.86
miR-18a	1.95	1.35	6.46
miR-18b	3.13	2.23	7.01
miR-20a	4.09	3.45	4.06
miR-20b	3.88	2.52	9.64
miR-21	−3.47	−3.78	−5.04
miR-22	4.30	2.38	4.41
miR-23b	−1.51	0.38	−1.09
miR-24	0.24	0.16	1.03
miR-26a	−2.38	−1.06	−1.53
miR-33a	6.78	6.10	8.13
miR-92a	ND*	−4.35	−0.70
miR-93	2.79	1.99	4.83
miR-96	6.48	4.04	5.81
miR-99a	1.84	1.36	1.96
miR-100	−1.24	0.91	0.89
miR-101	4.12	2.68	6.02
miR-103	1.96	1.15	3.99
miR-106b	2.10	1.72	5.96
miR-122	7.64	6.29	18.37
miR-124	0.48	−1.06	8.14
miR-125a-5p	−1.32	−0.34	−0.39
miR-125b	−4.01	−4.06	−3.12
miR-126	−0.04	−1.52	−0.50
miR-127-5p	4.50	3.10	13.88
miR-128a	3.99	4.42	4.09
miR-129-5p	−2.74	−4.04	6.80
miR-130a	1.16	−0.30	10.36
miR-132	1.68	0.45	2.05
miR-133b	4.43	3.60	17.30
miR-134	2.44	1.22	5.50
miR-137	5.22	4.34	10.05
miR-141	5.37	5.28	6.42
miR-142-3p	5.81	4.76	5.90
miR-142-5p	4.08	2.23	10.21
miR-146a	2.33	2.39	2.67
miR-146b-5p	3.61	2.79	0.46
miR-150	1.57	2.20	1.19
miR-155	5.85	4.67	4.72
miR-181a	3.80	2.59	6.15
miR-182	4.99	7.73	3.50
miR-183	4.72	5.17	4.29
miR-185	2.19	1.08	6.29
miR-192	2.16	0.75	6.06
miR-194	5.69	6.02	6.90
miR-195	−0.61	−0.45	−0.55
miR-196a	2.42	1.87	4.80
miR-205	0.30	0.13	−0.22
miR-206	6.18	1.47	10.57
miR-208	6.17	5.30	9.37
miR-210	−3.11	−4.50	6.75
miR-214	2.22	3.56	3.22
miR-215	1.00	−0.87	17.88
miR-218	3.85	2.64	2.21
miR-219-5p	3.30	2.18	10.09
miR-222	2.85	2.66	4.53
miR-223	1.28	0.15	0.87
miR-301a	4.11	2.90	15.12
miR-302a	5.21	4.02	13.05
miR-302c	7.16	6.81	16.00
miR-345	−0.62	−1.46	6.53
miR-370	2.22	1.22	8.77
miR-371-3p	9.74	8.85	11.20
miR-375	−1.41	−2.47	5.18
miR-378	1.85	1.54	4.21
miR-424	4.96	2.95	3.78
miR-452	5.89	4.88	8.29
miR-488	5.24	3.93	15.91
miR-498	4.46	3.14	16.82
miR-503	3.69	2.91	8.80
miR-518b	2.29	1.30	20.78
miR-520g	9.97	7.06	10.90

A mixture of equal amounts of miRNAs from 3 senile hemangioma, 3 venous malformation or 3 angiosarcoma were prepared, and miRNA expression profile in each tumor in vivo was evaluated using RT^2^ Profiler PCR Array. The raw threshold cycle (Ct) was normalized using the values of small RNA housekeeping genes. ΔΔCt (the raw Ct of each miRNA – Ct of small RNA housekeeping genes) were shown.

### Low mir-424 expression leads to MEK1- and cyclin E1-dependent cell proliferation in senile hemangioma

We tried to determine the role of mir-424 in the formation of senile hemangioma. According to miRNA target gene predictions of mir-424 using the TargetScan (version 5.1, http://www.targetscan.org/), a leading program in the field [27], we focused on MEK1 and cyclin E1 as the target genes of mir-424. MEK1 (mitogen-activated protein kinase kinase) is known as the upstream molecule of ERK and one of the most important mitogenic regulators [28]. Cyclin E1 is also implicated in the regulation of cell cycle [29]. We expected that decreased mir-424 expression causes increased cell proliferation via MEK1 and cyclin E1 in senile hemangioma.

**Figure 5:**
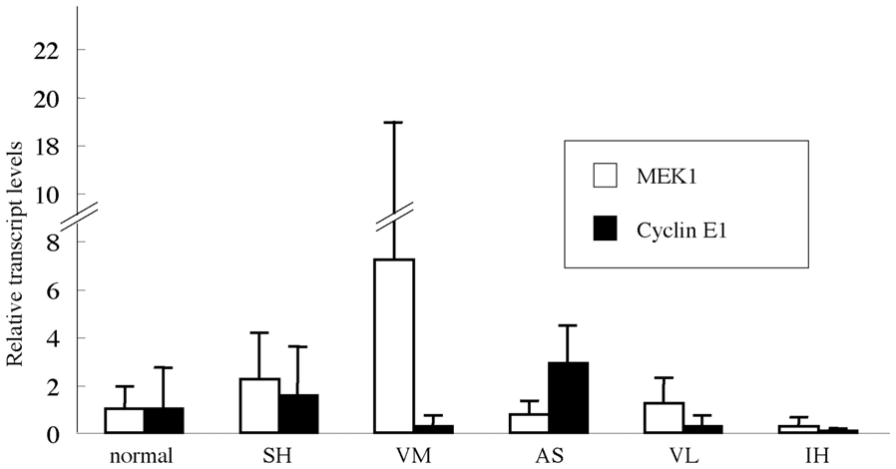
The transcript levels of MEK1 and cyclin E1 in senile hemangioma. Mean relative transcript levels of MEK1 (white bars) and cyclin E1 (black bars) in the tissues from normal skin (normal) or vascular anomalies such as senile hemangioma (SH), vascular malformation (VM), angiosarcoma (AS), venous lake (VL) and infantile hemangioma (IH) by real-time quantitative PCR. The transcript levels in normal skin were set at 1.

**Figure 6:**
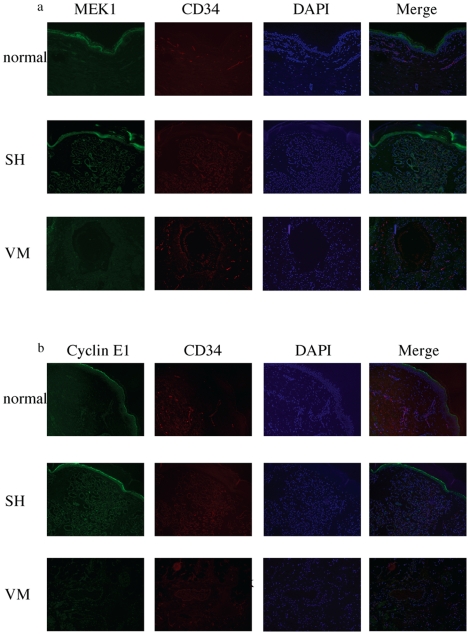
The immunoreactivity for MEK1 and cyclin E1 in senile hemangioma. Increased expression of MEK1 (a) or cyclin E1 (b) in ECs of senile hemangioma. Sections of normal skin or vascular anomalies were stained with antibodies against MEK1 or cyclin E1 (green) and CD34 (red). SH; senile hemangioma, VM; vascular malformation.

Then, we examined the mRNA levels of MEK1 or cyclin E1 in the tissue section. Although there was no significant difference in the mRNA levels between the section of senile hemangioma and those of other vascular anomalies (Fig. 5), protein expression of MEK1 (Fig. 6a) or cyclin E1 (Fig. 6b) on the proliferated vessels of senile hemangioma was increased compared to normal skin or venous malformation. To note, because CD34 (red) is expressed in cell surface whereas MEK1 and cyclin E1 (green) is in cytoplasm or nucleus, they did not co-localize [30], [31]. Considering that miRNAs usually inhibit translation of their target genes and do not cause degradation of the target transcript, our results indicate that mir-424 down-regulates MEK1 and cyclin E1 at the translation levels without altering mRNA levels, and that decreased mir-424 results in the overexpression of MEK1 and cyclin E1 in senile hemangioma.

**Figure 7:**
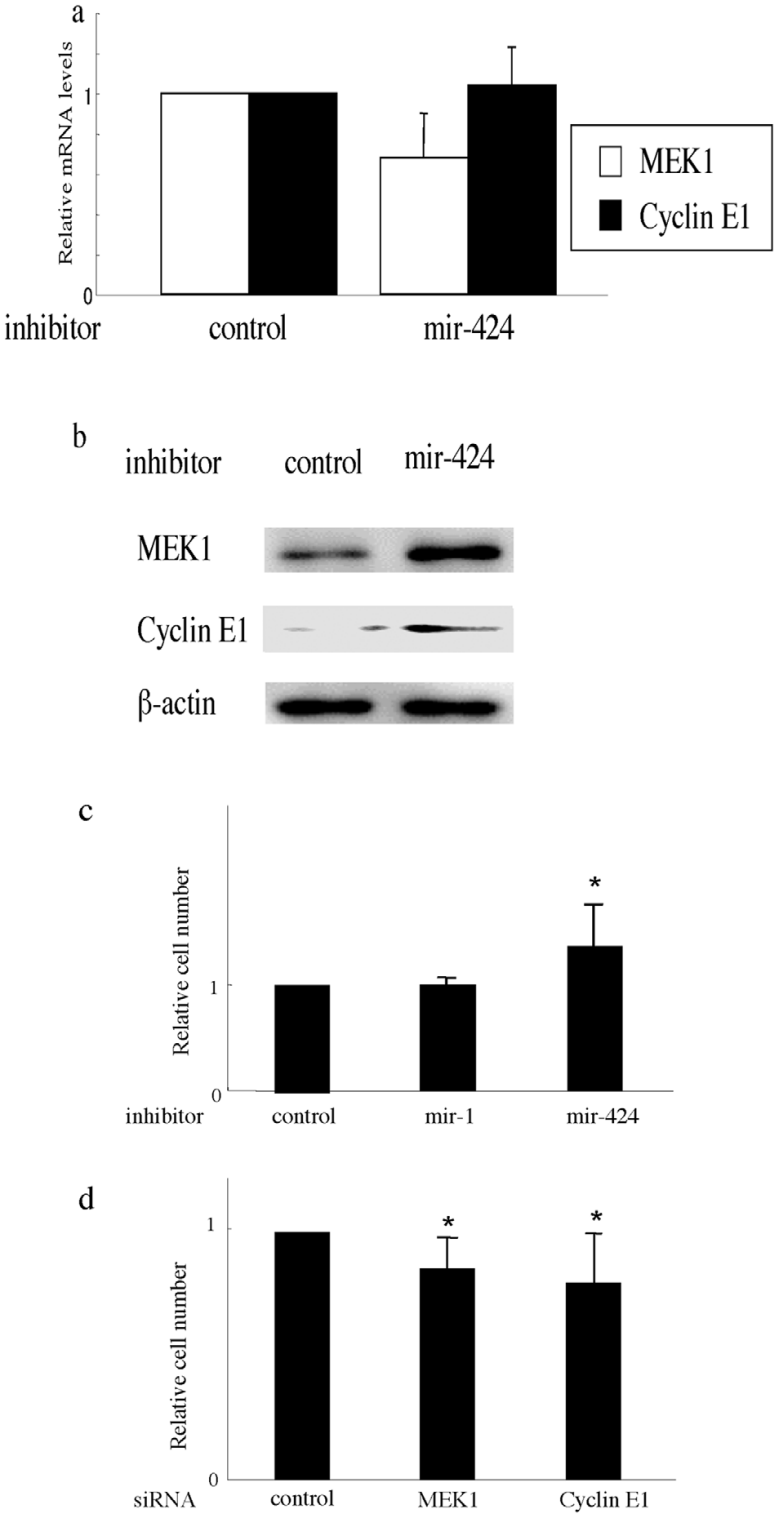
The association of mir-424 with MEK1 or cyclin E1. (a, b) mir-424 inhibitor induces the protein expression of MEK1 and cyclin E1 but not mRNA expression in human dermal microvascular ECs. Cells were transfected with the control inhibitor or mir-424 inhibitor for 48 hours. (a) Relative amounts of transcripts (normalized with GAPDH) determined in total RNA with quantitative PCR. Error bars represent SD of +1. (b) Lysates from cells transfected with control or mir-424 inhibitor were subjected to immunoblotting with antibody for MEK1 or cyclin E1. The same membrane was then stripped and reprobed with anti β-actin antibody as a loading control. (c, d) mir-424/MEK1/cyclin E1 pathway altered the cell proliferation activity. (c) HDMECs at a density of 5×10^3^ cells/well in 24-well culture plates were transfected with control inhibitor or inhibitors specific for mir-424 or mir-1. After 48 hours, the number of cells was counted with a Colter® Particle Counter (Beckman Coulter, Fullerton, CA) as described in ‘Materials and Methods’. The value in the cells transfected with control inhibitor were set at 1. The mean and SD from 3 separate experiments are shown. * P<0.05 in comparison to the value in the cells transfected with control inhibitor. (d) HDMECs at a density of 1×10^4^ cells/well in 24-well culture plates were transfected with control siRNA or siRNA specific for MEK1 or cyclin E1. After 72 hours, the cell number was counted as described in Fig. 7c.

To further investigate the association of mir-424 with MEK1 or cyclin E1, normal human dermal microvascular ECs (HDMECs) were transfected with mir-424 inhibitor and the expression of MEK1 and cyclin E1 was evaluated. The inhibition of mir-424 in vitro resulted in the increase of protein expression of MEK1 or cyclin E1, while mRNA levels were not significantly affected by the inhibitor (Fig. 7a and b), which is consistent with above result in vivo (Fig. 5 and 6).

Lastly, we determined whether mir-424 can be involved in the regulation of proliferative activity of ECs via MEK1 and cyclin E1. Specific inhibitor of mir-424 induced the cell proliferation significantly, whereas mir-1, which was also decreased in senile hemangioma by miRNA array analysis (see Table 2), did not (Fig. 7c). On the other hand, the transfection of siRNA for MEK1 or cyclin E1 led to slight but significant decrease in cell number (Fig. 7d). Taken together, decreased mir-424 expression and increased levels of MEK1 or cyclin E1 in senile hemangioma may cause abnormal cell proliferation in the tumor.

## Discussion

In this study, we have presented two major findings.

First, we showed that senile hemangioma and venous lake is likely to be categorized in vascular tumor and vascular malformation, respectively. The vessels of senile hemangioma are reported to be characterized by both proliferation and dilation [32]–[34]. Thus, it seems to be still unclear whether the tumor is either vascular tumor or malformation. The generic term ‘hemangioma’, classically used as a synonym of ‘vascular tumor’ in the classification above, has been frequently and incorrectly used by non-expert clinicians to describe different kinds of vascular anomalies. Confusion due to the persistent use of inappropriate and misleading terms still occurs: “hemangioma” simplex or cavernous “hemangioma” are terms still in use to name a capillary malformation or venous malformation, respectively. Our study may contribute to further understanding of the classification of vascular anomalies. On the other hand, venous lake are known to consists of dilated preexisting vessels, but there has been no obvious definition [35]. We showed that venous lake is similar to venous malformation, but the smooth muscle cells were maintained in venous lake. Further studies should be needed to clarify the cause of vessel dilation in this tumor.

Second, we found that mir-424 level was significantly reduced in senile hemangioma compared to normal skin and other vascular anomalies. mir-424 regulated cell proliferation via the silencing of the expression of MEK1 and cyclin E1. Recently, several miRNAs including let7-f, miR-27b and mir-130a have been implicated in angiogenesis [14], [15]. This is the first report implicating mir-424 in the angiogenic process and identifying MEK1 and cyclin E1 as the true target genes of mir-424. MEK1 and cyclin E1 may belong to the same signaling pathway to regulate cell cycle, because MEK1 is the up-stream molecule of ERK and cyclin E1 is down-stream target of ERK [36], [37]. Thus, mir-424/MEK1/cyclinE1 pathway may be able to regulate cell proliferation effectively. The effect of mir-424 inhibitor or siRNA for MEK1 or cyclin E1 on cell proliferation was significant, but moderate (20–40%, Fig. 7). However, for example, proliferative activity of ECs isolated from infantile hemangioma was increased only by 30–40%, compared with those of HDMECs [38], indicating that such modest increase of cell proliferation in vitro can cause hemangioma formation in vivo.

As reported previously, Val-Bernal et al. concluded that senile hemangiomas is a tissue overgrowth composed of mature vessels, lined by ECs with virtually no turnover [39]. Our results may sound contradictory to the report. However, this difference may be explained by the natural history of this tumor: Donsky et al. reported that microscopic examination of senile hemangioma in the early stage showed numerous newly formed capillaries and later these capillaries dilate [40]. The tumor in the previous report by Val-Bernal et al. seems to be late stage, because of the increased dilated vessels. Proliferative activation of ECs may be specific to early stage and it may disappear at the late stage.

Senile hemangioma may be the good model for cutaneous angiogenesis. Investigation of senile hemangioma and the regulatory mechanisms of angiogenesis by miRNA in the aged skin may lead to new treatments using miRNA by the transfection into senile hemangioma.
